# Study on Suitability of *KOD* DNA Polymerase for Enzymatic Production of Artificial Nucleic Acids Using Base/Sugar Modified Nucleoside Triphosphates

**DOI:** 10.3390/molecules15118229

**Published:** 2010-11-12

**Authors:** Masayasu Kuwahara, Yuuki Takano, Yuuya Kasahara, Hiroki Nara, Hiroaki Ozaki, Hiroaki Sawai, Akio Sugiyama, Satoshi Obika

**Affiliations:** 1 Graduate School of Engineering, Gunma University, 1-5-1 Tenjin-cho, Kiryu, Gunma 376-8515, Japan; 2 Tsuruga Institute of Biotechnology, Toyobo Co., Ltd., 10-24 Toyo-cho, Tsuruga, Fukui 914-0047, Japan; 3 Graduate School of Pharmaceutical Sciences, Osaka University, Osaka 565-0871, Japan

**Keywords:** *KOD* DNA polymerase, artificial nucleic acids, modified nucleoside triphosphates

## Abstract

Recently, *KOD* and its related DNA polymerases have been used for preparing various modified nucleic acids, including not only base-modified nucleic acids, but also sugar-modified ones, such as bridged/locked nucleic acid (BNA/LNA) which would be promising candidates for nucleic acid drugs. However, thus far, reasons for the effectiveness of *KOD* DNA polymerase for such purposes have not been clearly elucidated. Therefore, using mutated *KOD* DNA polymerases, we studied here their catalytic properties upon enzymatic incorporation of nucleotide analogues with base/sugar modifications. Experimental data indicate that their characteristic kinetic properties enabled incorporation of various modified nucleotides. Among those *KOD* mutants, one achieved efficient successive incorporation of bridged nucleotides with a 2′-ONHCH_2_CH_2_-4′ linkage. In this study, the characteristic kinetic properties of *KOD* DNA polymerase for modified nucleoside triphosphates were shown, and the effectiveness of genetic engineering in improvement of the enzyme for modified nucleotide polymerization has been demonstrated.

## 1. Introduction

To diversify the ability and enhance the nuclease resistance of functional nucleic acids like ribozymes and aptamers [1,2,3,4], enzymatic production of various modified nucleic acids has been investigated [5,6,7,8,9,10,11,12,13,14,15]. This is because those functional nucleic acids can be created by a random screening method, which involves enzymatic amplification of selected nucleic acids as a key step. We first demonstrated that *KOD Dash* DNA polymerase is very suitable for amplifying modified DNA by polymerase chain reaction (PCR) using the C5-substituted thymidine analogue [16]. Until now, many types of DNA polymerases were evaluated, and some of them, *e.g*. *Vent(exo-)*, *Pwo* and *Phusion High-Fidelity*, were found to be useful for enzymatic production of artificial nucleic acids. However, a series of experimental results from our laboratories indicated that *KOD Dash* DNA polymerase was more tolerant than any of the other aforementioned polymerases, not only towards base-modification, but also sugar-modification, and thus would be excellent for this purpose [17,18,19,20]. This is further supported by recent reports [21,22] regarding enzymatic production of bridged/locked nucleic acid (BNA [23,24]/LNA [25]) using *KOD* DNA polymerase. Furthermore, *KOD* DNA polymerase was often reported as the only one working for long PCR with modified nucleoside triphosphates [26,27].

**Figure 1:**
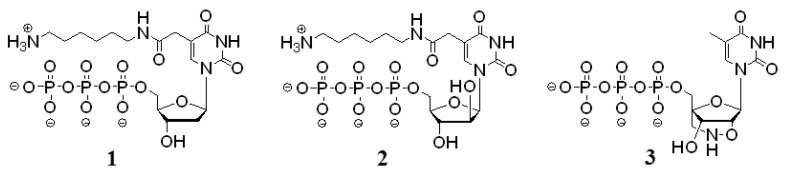
Chemical structures of the thymidine 5′-triphosphate analogues used in this experiment.

*KOD Dash* DNA polymerase is a mixture of wild-type *KOD* DNA polymerase and *KOD(exo-)* DNA polymerase having no or less 3′,5′ exonuclease activity. The wild-type *KOD* DNA polymerase belongs to the evolutional family B, and was originally isolated from the hyperthermophilic archaeon, *Pyrococcus kodakaraensis* [28]. Here, we have prepared eight *KOD* mutants (*KOD*1–8) [29,30], among which four mutants (*KOD*1–3, 8) have no or less 3′,5′ exonuclease activity, by genetically engineering *KOD* DNA polymerase, then assessed and compared the catalytic properties of those mutants, using thymidine analogues **1**–**3** (Figure 1) as substrates. Wild-type *KOD* DNA polymerase (Toyobo), *KOD Dash* DNA polymerase (Toyobo), and *Vent(exo-)* DNA polymerase (New England Biolabs) were also used as reference materials; *Vent(exo-)* is a genetically engineered form of the native DNA polymerase, which also belongs to family B, from *Thermococcus litoralis* [31,32].

## 2. Results and Discussion

First, we performed modified nucleotide standing-start experiments under conditions that achieve a single completed hit using template DNA T1 (5′- CCG AAT AAA GGT ATG GCC TGT TCG CTA ATC C -3′) and 5′-(6-FAM)-labelled primer P1 (5′- GGA TTA GCG AAC AGG CCA TAC CTT -3′). Four *KOD* mutants (*KOD*1–3, 8) and *Vent(exo-)* DNA polymerase were used as the enzyme, and thymidine analogues **1** and **2**, thymidine-5′-triphosphate (TTP) and 2′-deoxycytidine-5′-triphospate (dCTP), were used as the substrates. The reaction was performed at 40°C because the reactions proceeded too fast to be monitored at the optimal temperature of the enzyme (~75°C) [33]. All reactions were performed under the same enzyme concentration of 0.0125 U/μL. The apparent values for *K*_m_ and *V*_max_ were obtained from the initial velocities of the primer extension reactions, and then the apparent insertion efficiency (*V*_max_/*K*_m_) of each substrate triphosphate usage was calculated. The relative apparent insertion efficiency (accuracy) was defined as the ratio of modified analogue insertion to TTP correct insertion efficiencies (*V*_max_/*K*_m_)_analogue_/(*V*_max_/*K*_m_)_TTP_, or that of dCTP misinsertion to TTP correct insertion efficiencies (*V*_max_/*K*_m_)_dCTP_/(*V*_max_/*K*_m_)_TTP_ as shown in Table 1. 

In incorporation of thymidine analogues **1** and **2**, all *KOD* mutants used showed apparent *K*_m_ values larger than that for natural TTP; those values were about 1.7- to 6.4-fold in the cases of the base-modified analogue **1**, and were about 2.1- to 13-fold in the cases of the base- and sugar-modified analogue **2**. On the other hand, *Vent(exo-)* DNA polymerase showed *K*_m_ values for those two analogues that were almost same as that for TTP. In misincorporation of dCTP, apparent *K*_m_ values of *KOD* mutants were only 3.2- to 32-fold larger than that for TTP as a correct incorporation substrate, while that of *Vent(exo-)* was about 580-fold larger than that for TTP. However, relative insertion efficiency (accuracy), in other words, substrate specificity of polymerase of when insertion of thymidine nucleotide is the correct incorporation, were almost the same between *KOD* mutants and *Vent(exo-)* DNA polymerase. This is because *KOD* mutants provide larger *K*_m_ and *V*_max_ in the cases of analogue incorporation, and smaller *K*_m_ and *V*_max_ in the cases of the misincorporation, compared with *Vent(exo-)* DNA polymerase. As a result, only slight differences in the ratios of *K*_m_ to *V*_max_ were seen between *KOD* mutants and *Vent(exo-)* DNA polymerase. This catalytic property of *KOD* mutants would be favourable for enzymatic production of artificial nucleic acids, that is, large *V*_max_ values of *KOD* mutants in analogue incorporation per misincorporation indicate that the reaction of analogue incorporation could progress much faster than that of dCTP misincorporation under sufficiently high substrate concentrations. 

We then investigated successive incorporations of modified nucleotide by primer extension reactions using bridged nucleotide **3**, because bridged/locked nucleic acids (BNA/LNAs) would be promising candidates for nucleic acid drugs [34,35]. We have previously reported [36] that incorporation of this type of bridged nucleotide (2′,4′-BNA^NC^) [37] is more difficult than that of the prototype 2′-*O*,4′-*C*-methylene bridged/locked nucleotide (BNA/LNA), which can be incorporated successively using wild-type *KOD* DNA polymerase [23]. However, 2′,4′-BNA^NC^–type nucleotides possess excellent nuclease resistance much superior to the prototype [38,39]; therefore, its applications in random screening of nucleic acid enzymes and aptamers, which would require efficient enzymatic incorporation of modified nucleotides, are highly anticipated. In this experiment, template DNA T2 (5′- CCG AAA AAA GGT ATG GCC TGT TCG CTA ATC C -3′), 5′-(6-FAM)-labeled primer P2 (5′- GGA TTA GCG AAC AGG CCA TAC CTT T -3′) and eight *KOD* mutants (*KOD*1–8) and *Vent(exo-)* DNA polymerase were used. 

**Figure 2:**
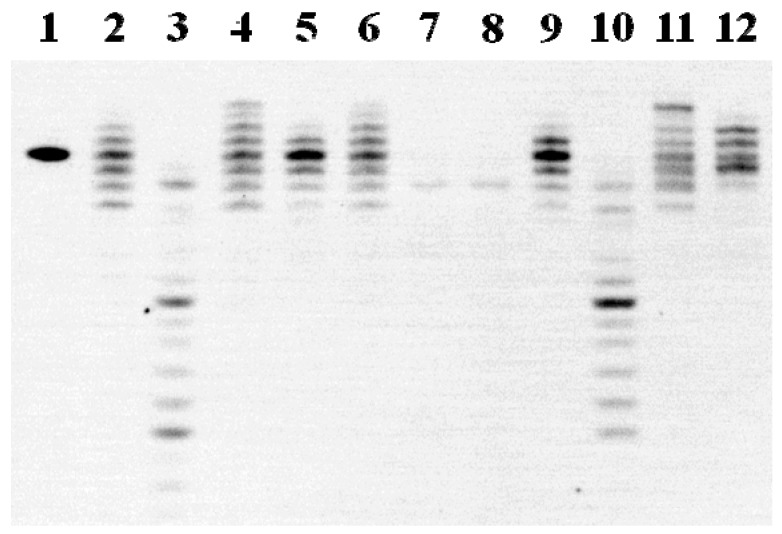
Successive incorporation of 2′,4′-bridged nucleotides using analogue **3** with various DNA polymerases; *KOD Dash* (lane 2), wild type *KOD* (lane 3), *KOD*1 (lane 4), *KOD*2 (lane 5), *KOD*3 (lane 6), *KOD*4 (lane 7), *KOD*5 (lane 8), *KOD*6 (lane 9), *KOD*7 (lane 10), *KOD*8 (lane 11), and *Vent(exo-)* (lane 12). Primer extension reactions were performed for 1 h at 74°C under enzyme concentration of 0.4 U/μL. Primer P2 only migrated in lane 1.

As shown in Figure 2, wild-type *KOD* DNA polymerase, *KOD*4, *KOD*5 and *KOD*7 exhibited the strong 3′,5′ exonuclease activity [40], and did not provide any elongated products, while the other polymerases provided some elongated products. Among these, *KOD*8 mainly provided a longer elongated product; it would be four-successive incorporations although three-successive incorporated products must be provided as the full-length product under the condition using those materials. This may have occurred because of DNA template slippage caused by conformation of DNA/BNA helix different from the natural DNA/DNA helix between primer and template strands [41]. Note that the efficient multiple-successive incorporation of bridged analogue **3** under severe conditions such as without any additives, *e.g.* manganese chloride and betaine [42,43], could be made by one of *KOD* mutants assessed, *KOD*8.

## 3. Experimental

### 3.1. Synthesis of modified nucleoside triphosphate analogs and their intermediates

**Scheme 1:**
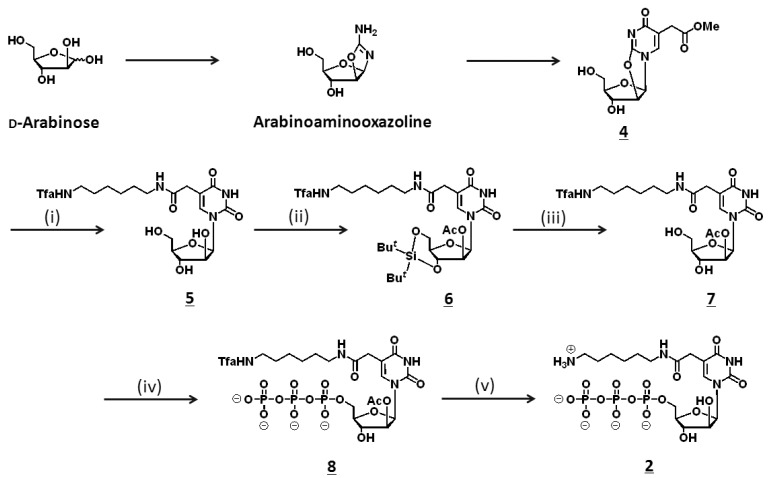
Synthesis of thymidine analogue **2**.

The modified nucleoside triphosphates **1** and **3** were synthesized according to our previous reports [16,36]. The synthetic routes of the modified nucleoside triphosphate **2** and its intermediates are shown in Scheme 1. NMR Spectra were recorded on a JEOL JNM-AL300 FT-NMR spectrometer. Mass spectral analysis for nucleoside analogues was accomplished on an MDS-Sciex API-100 spectrometer (Applied Biosystems) under atmospheric pressure ionization conditions. Thin-layer chromatography was performed using silica gel 60 F_254_ plates (Merck). Reversed-phase high-performance liquid chromatography (HPLC) was performed using a JASCO Gulliver system with UV detection at 260 nm and a packed Wakosil 5C18 (*ϕ* 4.6×250 mm; Wako) or TSKgel ODS-80Ts (*ϕ* 20×250 mm; Tosoh) column. Silica gel 60 (Kanto Chemical; 40–50 μm) was used for normal-phase column chromatography, and Wakosil 40C18 (Wako) was used for reversed-phase column chromatography. Reversed-phase medium-pressure liquid chromatography (MPLC) was performed using an YFLC-Wprep system (Yamazen) with a glass column (*ϕ* 33×250 mm) filled with Wakosil 40C18 (Wako). Ion exchange column chromatography was performed using an ECONO system (Bio-Rad) with a glass column (*ϕ*25×500 mm) filled with diethylaminoethyl (DEAE) A-25-Sephadex (Amersham Biosciences).

Nucleoside **5** was synthesized as follows: dimethylaminopyridine (118 mg; 0.97 mmol; 0.1 eq) and hexamethylenediamine (5.0 g; 97.0 mmol; 10 eq) were added to a stirred solution of nucleoside **4** [44] (2.9 g; 9.7 mmol) in dry methanol (40 mL). The reaction mixture was stirred overnight at 50°C. After evaporation, the residue was dissolved in methanol (10 mL), 2N NaOH aq. (25 mL) was added, and the mixture was stirred. After 36 h, the reaction mixture was neutralized with aq. 2N HCl. After evaporation, the residue was dissolved in dry methanol (50 mL), and then trifluoroacetic acid ethyl ester (13.8 mg; 97.0 mmol; 10.0 eq) and triethylamine (7.4 mg; 72.8 mmol; 7.5 eq) were added dropwise, and the mixture was stirred for 18 h. After evaporation, the residue was purified by flash column chromatography on silica gel eluting with 5% to 15% methanol in chloroform. To remove the excess triethylamine, the residue was purified by reversed-phase MPLC with a linear gradient of 0% to 50% methanol in 0.1% trifluoroacetic acid aq. to give the nucleoside **5** (1.075 g; 23%): Positive ion ES-MS *m*/*z* [assignment] 519.3 [(M+Na)^+^], 535.3 [(M+K)^+^]; ^1^H-NMR (300 MHz, CDCl_3_) δ 7.81 (s, 1H), 6.13 (d, 1H), 4.87 (s, 2H), 4.58 (s, 1H), 4.18 (t, 1H), 4.07 (t, 1H), 3.90 (m, 1H), 3.81 (t, 1H), 3.14 (m, 4H), 1.54 (m, 4H), 1.34 (m, 4H); ^1^H-NMR (300 MHz, CD_3_OD) δ 7.81 (s, 1H, C_6_*H*), 6.13 (d, J = 4.5 Hz, 1H, C_1__′_*H*), 4.16 (m, 1H, C_2__′_*H*), 4.07 (m, 1H, C_3__′_*H*), 3.91 (m, 1H, C_4__′_*H*), 3.80 (m, 2H, C_5__′_*H*_2_), 3.29 (m, 2H, C_5_*H*_2_), 3.19 (m, 4H, C(=O)NHC*H*_2_(CH_2_)_4_C*H*_2_NHTfa), 1.55 (m, 4H, C(=O)NHCH_2_C*H*_2_ (CH_2_)_2_C*H*_2_CH_2_ NHTfa), 1.35 (m, 4H, C(=O)NH(CH_2_)_2_(C*H*_2_)_2_(CH_2_)_2_NHTfa).

Nucleoside **6** was synthesized as follows: nucleoside **5** (1.075 g; 2.2 mmol) was dried in a flask under vacuum overnight. Dry *N*,*N*-dimethylformamide (20 mL) was added to the flask under argon, and the solution was cooled to 0°C. Then, di-*tert*-butylsilyl-*bis*(trifluoromethanesulfonate) (0.77 mL; 2.4 mmol; 1.1 eq) was added, and the mixture was stirred at 0°C for 1h. Imidazole (750 mg; 11.0 mmol; 5.0 eq) was added to the reaction mixture and stirred at room temperature for 1 h. Pyridine (3.0 mL) and acetic anhydride (1.67 mL; 17.6 mmol; 8.0 eq) were then added, and stirred for 1 h. After evaporation, the residue was dissolved in dichloromethane and washed with water. The organic layer was dried over MgSO_4_, filtered, and concentrated in *vacuo*. Flash column chromatography on silica gel eluting with acetic ether afforded the nucleoside **6** (785 mg, 53%): Positive ion ES-MS *m*/*z* [assignment] 701.4 [(M+Na)^+^]; ^1^H-NMR (300 MHz, CDCl_3_) δ 7.58 (s, 1H), 6.45 (s, 1H), 5.28 (t, 1H), 4.44 (q, 1H), 4.22 (m, 1H), 4.07 (q, 2H), 3.35 (m, 1H), 3.35 (q, 1H), 3.20 (q, 4H), 2.04 (s, 3H), 1.49 (t, 4H), 1.35 (t, 4H), 1.00 (s, 18H). ^1^H-NMR (300 MHz, CDCl_3_) δ 7.23 (s, 1H, C_6_*H*), 6.45 (d, 1H, C_1__′_*H*), 5.25 (t, 1H, C_2__′_*H*), 4.43 (q, 1H, C_3__′_*H*), 4.08 (m, 2H, C_5__′_*H*_2_), 3.71 (m, 1H, C_4__′_*H*), 3.34 (m, 2H, C_5_*H*_2_), 3.19 (m, 4H, C(=O)NHC*H*_2_(CH_2_)_4_C*H*_2_NHTfa), 2.04 (s, 3H, OC(=O)C*H*_3_), 1.53 (m, 4H, C(=O)NHCH_2_C*H*_2_(CH_2_)_2_ C*H*_2_CH_2_NHTfa), 1.33 (m, 4H, C(=O)NH(CH_2_)_2_(C*H*_2_)_2_(CH_2_)_2_NHTfa), 1.10 (s, 9H, Si(*Bu^t^*)_2_), 1.01 (s, 9H, Si(*Bu^t^*)_2_).

Nucleoside **7** was synthesized as follows: nucleoside **6** (785 mg; 1.16 mmol) was dried in a flask under vacuum overnight. Dry dichloromethane (10.8 mL) was added to the flask under argon, and dry pyridine (2.16 mL) and pyridinium poly(hydrogenfluoride) (360 μL) were added, and the reaction mixture was stirred at room temperature for overnight. After evaporation, the residue washed with diethyl ether, and then purified by flash column chromatography on silica gel eluting with 95:5 dichloromethane-methanol to give the nucleoside **7** (432 mg; 69%): Positive ion ES-MS *m*/*z* [assignment] 561.3 [(M+Na)^+^]; ^1^H-NMR (300 MHz, CD_3_OD) δ 7.79 (s, 1H, C_6_*H*), 6.26 (d, J = 4.9 Hz, 1H, C_1__′_*H*), 5.26 (m, 1H, C_2__′_*H*), 4.25 (m, 1H, C_3__′_*H*), 3.89 (m, 1H, C_4__′_*H*), 3.84 (m, 2H, C_5__′_*H*_2_), 3.29 (m, 2H, C_5_*H*_2_), 3.18 (m, 4H, C(=O)NHC*H*_2_(CH_2_)_4_C*H*_2_NHTfa), 2.01 (s, 3H, OC(=O)C*H*_3_), 1.54 (m, 4H, C(=O)NHCH_2_C*H*_2_(CH_2_)_2_C*H*_2_CH_2_NHTfa), 1.35 (m, 4H, C(=O)NH(CH_2_)_2_(C*H*_2_)_2_(CH_2_)_2_NHTfa).

Nucleoside triphosphate **8** was synthesized as follows: nucleoside **7** (106 mg; 0.2 mmol) and *N*,*N*,*N′*,*N′*-tetramethyl-1,8-naphthalenediamine (Proton Sponge^®^; 64 mg; 0.3 mmol; 1.5 eq) were dried in a flask under vacuum overnight. Trimethyl phosphate (1.5 mL) was added to the flask under argon, and the solution was cooled to 0°C. Distilled phosphorus oxychloride (23 μL; 0.24 mmol; 1.2 eq) was then added dropwise using a micro syringe, and the reaction mixture was stirred at 0°C. After 45 min, *n*-tributylamine (191 μL; 0.8 mmol; 4.0 eq) and *n*-tributylamine pyrophosphate (2.06 mL of a 0.5 M solution in DMF, 1.0 mmol, 5.0 eq) were added at 0°C, and the reaction mixture was stirred at room temperature for 1 h, then the reaction was quenched with water. The solvents were removed *in vacuo*, and the residue was dissolved in water. The product was purified using a Sephadex DEAE A-25 column with a linear gradient of 0.05–1.0 M triethylammonium bicarbonate buffer (pH 8). Fractions containing **8** were combined and evaporated under reduced pressure, then purified by reversed-phase MPLC with a linear gradient of 0% to 70% acetonitrile in 10 mM triethylammonium acetate buffer (pH 7), to remove the excess pyrophosphate. Further purification was performed by reversed-phase HPLC (*ϕ*20 × 250 mm) with a linear gradient of 35% to 70% acetonitrile in 50 mM triethylammonium acetate buffer (pH 7) to give the nucleoside triphosphate **8** (36.7 μmol) in 19% yield from **7**: Negative ion ES-MS *m*/*z* [assignment] 776.8 [(M-H)^-^].

Nucleoside triphosphate **2** was synthesized as follows: to a stirred solution of triphosphate **8** (3.7 μmol) in water (1mL), 4N NH_4_OH aq. (1 mL) was added, and the mixture was stirred at room temperature for 2 h. The solvents were removed *in vacuo*, and the residue was dissolved in water. Purification was performed by reversed-phase HPLC (*ϕ*20×250 mm) with a linear gradient of 0% to 50% acetonitrile in 50 mM triethylammonium acetate buffer (pH 7) to give the nucleoside triphosphate **2** (1.1 μmol) in 30% yield from **8**: Negative ion ES-MS *m*/*z* [assignment] 639.1 [(M-H)^-^].

### 3.2. Preparation of mutated KOD DNA polymerases

Expression and purification of the *KOD* mutants used were performed basically according to the literatures [29,30]. 

### 3.3. Single nucleotide incorporation in primer extension reaction using KOD mutants and Vent(exo-) DNA polymerase

Reaction mixtures (8 μL) containing the primer (P1) at 0.5 μM, the template (T1) at 0.625 μM, one of the thymidine 5′-triphosphates (1, 2 or TTP) at 1 μM, 3 μM, 5 μM, 10 μM, 20 μM and 30 μM, or 2′-deoxycytidine-5′-triphosphate (dCTP) at 0.1 mM, 0.3 mM, 0.5 mM and 1 mM, and the reaction buffer supplied with the enzyme (at 1× concentration) were denatured at 95°C for 1 min with the thermal cycler, and then annealed at room temperature for 1 h. Subsequently, 2 μL of enzyme solution (0.125 U for *Vent(exo-)* and *KOD* mutants (*KOD*1-3, 8)) were added to the mixture, and the reaction tube was quickly placed in a thermoregulated bath and incubated at 40°C during the reaction. Reactions were monitored at 0, 1, 3, 7, 15, 25 and 60 min in the cases of the thymidine 5′-triphosphates (1, 2 and TTP), also at 0, 7, 15, 30, 50, 80, 120, and 180 min in the case of dCTP. After the reactions were started, the reaction tubes were removed from the bath sequentially, and immediately quenched by freezing in liquid nitrogen. The frozen reaction mixtures were then mixed with 40 mM EDTA (2 μL) containing 0.1% bromophenol blue and 7 M urea (12 μL) containing 3 mM EDTA, and then were melted into a homogeneous solution by vortexing. The sample solutions were resolved by denaturing PAGE, and gel images were recorded on the imager. The amount of reactant and products was measured from the intensity of each band with excitation at 488 nm to visualize the 5′-labelled fluorophore. The decrease of the primer ratio (%) was obtained from band intensities of the primer and its elongated products. The data of the time-dependent decrease were fit to hyperbolic saturation curves by the least squares method using OriginPro ver.8.

### 3.4. Successive incorporation of 2′,4′-bridged nucleotides using analogue 3 with various DNA polymerases

Reaction mixtures (8 μL) containing the primer (P2) at 0.5 μM, the template (T2) at 0.625 μM, 2,4-bridged nucleotide analogue **3** at 200 μM, and the reaction buffer supplied with the enzyme (at 1× concentration) were denatured at 95°C for 1 min with the thermal cycler and then annealed at room temperature for 1 h. Subsequently, 2 μL of enzyme solutions [4 U for *Vent(exo-), KOD Dash*, wild-type *KOD* and *KOD* mutants (*KOD*1-8)] were added to the mixture, and the reaction tube was quickly placed in a thermoregulated bath and incubated at 74°C during the reaction at 60 min. After the reactions were started, the reaction tubes were removed from the bath, and immediately quenched by freezing in liquid nitrogen. The frozen reaction mixtures were then mixed with 40 mM EDTA (2 μL) containing 0.1% bromophenol blue and 7 M urea (12 μL) containing 3 mM EDTA, and then were melted into a homogeneous solution by vortexing. The sample solutions were resolved by denaturing PAGE, and gel images were recorded on the imager. The amount of reactant and products was measured from the intensity of each band with excitation at 488 nm to visualize the 5′-labelled fluorophore. 

## 4. Conclusions

We have found that *KOD* mutants provide large *V*_max_ values in the correct analogue incorporations, but relatively small *V*_max_ values in misincorporation; on the other hand, other polymerases such as DNA polymerase α involving *Vent(exo-)* DNA polymerase could mainly distinguish correct incorporation from misincorporation through differences in the *K*_m_ values [45]. This nature of *KOD* DNA polymerase is considered to enable incorporation of modified nucleotides even with a unique chemical structure like the aforementioned BNA. Thus, catalysts of enzymatic production of artificial nucleic acids with excellent fidelity and tolerance for various chemical modifications will be expected through further improvements based on amino acid sequence of *KOD* DNA polymerase.

## Figures and Tables

*Reagents and Conditions*: (i) hexamethylenediamine, DMAP, 50°C, 1 h; 2N NaOH aq., r.t., 36 h; trifluoroacetic acid ethylester, TEA, 18 h; (ii) di-*tert*-butylsilyl-*bis*(trifluoro- methanesulfonate), DMF, 0 °C, 1 h; imidazole, r.t., 1 h; pyridine, acetic anhydride, r.t., 1 h; (iii) pyridinium poly(hydrogenfluoride), r.t., overnight; (iv) POCl_3_, *N*,*N*,*N′*,*N′*-tetramethyl-1,8-naphthalendiamine, trimethyl phosphate, 0°C, 45 min; *n*-tributylamine pyrophosphate, DMF, r.t., 1 h; (v) 4N NH_4_OH aq., r.t., 2 h.

**Table 1:** Modified/natural nucleotide incorporation in primer extension reaction using *KOD* mutants and *Vent(exo-)* DNA polymerase. *^a^*

DNA polymerase	dNTP	*V*_max_ (min^–1^)	*K*_m_ (mM)	Insertion efficiency *V*_max_/*K*_m_	Accuracy*^b^*
*KOD*1	T	1.8	0.021	88	1
	**1**	11	0.14	81	0.92
	**2**	0.10	0.26	3.8	0.043
	C	0.074	0.66	0.11	0.0013
*KOD*2	T	2.1	0.035	60	1
	**1**	4.0	0.083	48	0.80
	**2**	0.32	0.099	3.3	0.055
	C	0.019	0.11	0.17	0.0029
*KOD*3	T	2.3	0.047	49	1
	**1**	4.4	0.081	55	1.1
	**2**	0.27	0.099	2.7	0.055
	C	0.033	0.70	0.048	0.00097
*KOD*8	T	9.5	0.063	150	1
	**1**	53	0.31	170	1.1
	**2**	2.4	0.38	6.3	0.041
	C	0.11	1.0	0.11	0.00073
*Vent(exo-)*	T	0.34	0.0030	110	1
	**1**	0.29	0.0027	110	0.95
	**2**	0.049	0.0044	11	0.099
	C	0.14	1.7	0.083	0.00073

*^a^*Experimental conditions are described in Experimental Section. *^b^*The initial rate relative apparent insertion efficiency (accuracy); (*V*_max_/*K*_m_)_analogue_/(*V*_max_/*K*_m_)_TTP_ and (*V*_max_/*K*_m_)_dCTP_/(*V*_max_/*K*_m_)_TTP_.
